# Exploring Human Papillomavirus 16 Long Control Region Variants in Paraguayan Women: Comparing Variant Diversity to Transcriptional Activity and Cervical Lesion Severity

**DOI:** 10.1155/ijm/2292032

**Published:** 2026-07-06

**Authors:** Pamela Mongelós, Magaly Martínez, Natalia Díaz, Valéria Talpe Nunes, Adriana Valenzuela Cáceres, Alexandra Mercado-Amarilla, Mabel Miranda, Laura Bernal, Elena Kasamatsu, Malvina Páez, Amalia Castro, María Isabel Rodriguez-Riveros, Hernan Barrios, Armando Baena, Maryluz Rol, Maribel Almonte, Rolando Herrero, Laura Sichero, Laura Mendoza

**Affiliations:** ^1^ Health Sciences Research Institute (IICS), National University of Asuncion (UNA), San Lorenzo, Paraguay; ^2^ Center for Translational Research in Oncology, Cancer Institute of the State of Sao Paulo (ICESP), Hospital of Clinic of the Faculty of Medicine of the University of Sao Paulo (HC FMUSP), São Paulo, Brazil; ^3^ Comprehensive Center for Precision Oncology, University of Sao Paulo, São Paulo, Brazil, usp.br; ^4^ Division of Cancer Epidemiology and Genetics, National Cancer Institute, National Institutes of Health, Bethesda, Maryland, USA, nih.gov; ^5^ Early Detection, Prevention and Infection Branch, International Agency for Research on Cancer, World Health Organization, Lyon, France, who.int; ^6^ Department of Noncommunicable Diseases, Rehabilitation and Disability, World Health Organization, Geneva, Switzerland, who.int; ^7^ Costa Rican Agency for Biomedical Research, San José, Costa Rica

**Keywords:** CIN2+ lesions, HPV16, long control region, Paraguayan women, transcriptional activity, variants

## Abstract

Human papillomavirus Type 16 (HPV16) variants are classified into four lineages (A–D) and 16 sublineages: A1–3 (European), A4 (Asian), B1–4 and C1–4 (African), and D1–4 (Asian‐American). This study investigated the genetic HPV16 variability through sequencing of the complete long control region (LCR), in 44 cervical samples from Paraguayan women, including 19 without cervical intraepithelial neoplasia grade 2 (<CIN2) and 25 with CIN2 or worse (CIN2+: 1 CIN2, 16 CIN3, and 8 invasive cancers). Sublineages A1 (81.8%), A4 (2.3%), D2 (2.3%), D3 (11.4%), and D4 (2.3%) were detected. A total of 48 point mutations were detected, of which 11 showed nominal association with CIN2+ (*p* < 0.05), although none remained statistically significant after correction for multiple comparisons. Notably, most of these mutations were predominantly observed in non‐A1–A3 variants. An exploratory association was observed between non‐A1–A3 variants and the presence of CIN2+ *(* *p* = 0.0065). Functional analysis revealed that the A4 variant, detected in a woman with cancer, exhibited the highest transcriptional activity (2.34 ± 0.78), followed by D3 (0.72 ± 0.21) and A1 (0.03 ± 0.02), corresponding to samples from women with CIN3 and without lesions, respectively. In conclusion, we report for the first time the detection of an A4 HPV16 variant in Paraguay, which exhibited the highest transcriptional activity among the variants analyzed. These findings suggest potential functional differences among HPV16 variants and are consistent with a possible relevance of non‐A1–A3 variants in cervical lesion severity. However, these results should be interpreted with caution as exploratory and hypothesis‐generating.

## 1. Introduction

Human papillomavirus (HPV) is a nonenveloped virus with a circular, double‐stranded DNA genome of approximately 8000 base pairs (*bp*) in length. The genome consists of three main regions: the early region, which contains open reading frames (ORFs) E1, E2, E4, E5, E6, and E7 involved in transcriptional regulation, replication, transformation, and immune evasion; the late region, composed of ORFs L1 and L2, which encode the structural capsid proteins; and the upstream regulatory region (URR), also known as the long control region (LCR, 7157–82 bp for HPV‐16), a noncoding segment that regulates viral transcription and replication through transcription factor binding sites [1, 2].

HPV belongs to the family *Papillomaviridae* and based on nucleotide identity of the *L1* gene, is classified into genera, species, types, and subtypes. A novel type differs from known types by more than 10% within the *L1* gene sequence, whereas subtypes show 2%–10% difference. Based on whole genome sequence, variants are further divided into lineages (1%–2% difference) and sublineages (0.5%–1%). Thirteen mucosal HPV types from the *Alphapapillomavirus* genus are considered high‐risk and are responsible for the majority of cervical cancer cases worldwide [3–5].

Globally, HPV16 accounts for approximately 62% of cervical cancer cases [6, 7]. In Paraguay, HPV16 is also the most frequently detected type in women with and without cervical lesions, as well as in invasive cervical cancer cases [8–11].

To date, HPV16 variants are classified into four lineages (A–D) and 16 sublineages: lineage A includes A1, A2, A3 (previously nominated European), and A4 (Asian); lineage B includes B1–B4 (African‐1); lineage C includes C1–C4 (African‐2); and lineage D includes D1 (North American), D2, D3 (Asian‐American), and D4 [12]. These variants display distinct geographic distributions and may differ in biological behaviors and oncogenic potential [13].

The LCR region is frequently used to study HPV16 intratype diversity and assign variants to lineages, as it is the most variable region of the viral genome. Functional differences between variants are often linked to sequence variations within the LCR, which affect transcription factor binding and may influence viral persistence and oncogenicity [1, 14, 15]. Despite growing evidence, the relationship between HPV16 variants, persistent infection, and lesion progression remains incompletely understood [16].

This study is aimed at characterizing and evaluating HPV16 variants in cervical samples from Paraguayan women participating in the ESTAMPA Paraguay Project [17]. Our findings are expected to contribute to novel data on the distribution of HPV16 variants in Paraguay in women with and without CIN2+ and to provide data on their transcription activity according to cervical lesion severity.

## 2. Materials and Methods

### 2.1. Study Design and Sample Collection

This was an observational, descriptive, cross‐sectional study. A total of 58 cervical exfoliated cell samples from HPV16‐positive Paraguayan women aged 30 to 64 years were included. Of these, 32 samples were from women without CIN2 and 26 from women with CIN2+ (1 CIN2, 17 CIN3, and 8 invasive cancers).

Samples were collected by trained clinical personnel using a Cervex‐Brush and stored at –80°C until processing. All specimens were obtained from the ESTAMPA‐Paraguay study, a multicenter cervical cancer screening study conducted between February 2014 and September 2017 [18].

Clinical data were accessible only to the principal investigators. Samples were anonymized prior to molecular and functional analyses, and all laboratory procedures, including sequencing and luciferase assays, were performed without knowledge of the clinical diagnosis associated with each sample.

### 2.2. PCR Amplification and Sequencing of the LCR

The complete HPV16 LCR was amplified in two overlapping fragments using previously described primers and conditions [14]. High‐fidelity amplification was performed using iProof High‐Fidelity DNA Polymerase (Bio‐Rad, United States).

For the first fragment (LCR1; approximately 460 bp, nt 7101–7560), primers LCR1F (5 ^′^‐ACCCACCACCTCATCTACCTCTACAA‐3 ^′^) and LCR1R (5 ^′^‐ATTTGGCACGCATGGCAAGCAGGAA‐3 ^′^) were used. The second fragment (LCR2; approximately 621 bp, nt 7465–180) was amplified using primers LCR2F (5 ^′^‐CATGCTTTTTGGCACAAAATGTGTTTT‐3 ^′^) and LCR2R (5 ^′^‐ATATCATGTATAGTTGTTTGCAGCTCT‐3 ^′^).

PCR reactions were carried out in a final volume of 50 *μ*L containing 1× iProof buffer, 0.2 mM dNTPs, 0.2 *μ*M of each primer, 0.02 U/*μ*L DNA polymerase, and 5 *μ*L of template DNA. Thermal cycling conditions consisted of an initial denaturation at 98°C for 3 min, followed by 35 cycles of denaturation at 98°C for 10 s, annealing at 57°C for 30 s, and extension at 72°C for 30 s, with a final extension at 72°C for 10 min.

PCR products were verified by electrophoresis on 1% agarose gels and purified using the Wizard SV Gel and PCR Clean‐Up System (Promega, United States), following the manufacturer′s instructions. Purified amplicons were quantified using the Qubit dsDNA HS Assay Kit (Thermo Fisher Scientific, United States), and samples with sufficient DNA concentration (> 50 ng/*μ*L) were selected for sequencing.

Purified PCR products (25 *μ*L) and corresponding primers (15 *μ*L at 100 *μ*M) were submitted for bidirectional Sanger sequencing (Macrogen Inc., Seoul, Korea).

Only samples yielding complete LCR sequences were included in downstream analyses.

### 2.3. Variant Identification and Phylogenetic Analysis

The LCR region (nt 7157–7906 and 1–82) was aligned with HPV16 reference sequences obtained from GenBank and the Papillomavirus Episteme (PaVE) database using MAFFT v7.

Single‐nucleotide polymorphisms (SNPs) were identified by systematically comparing each sequence against the HPV16 reference genome (HPV16REF_PaVE; GenBank accession K02718). SNP detection was performed using custom scripts implemented in R (R Core Team, 2025) [19]. These scripts parsed the multiple sequence alignment to extract nucleotide differences at each position relative to the reference sequence and generated mutation tables and presence/absence matrices for downstream analyses. Data processing and manipulation were performed using the R packages *Biostrings*, *dplyr*, *tidyr*, *stringr*, *purrr*, *read*r, and *tibble* [20–26].

Variant assignment to lineages and sublineages was determined based on phylogenetic clustering with reference sequences. Phylogenetic reconstruction was performed using BEAST v1.10.4, under the HKY + I + G substitution model and an uncorrelated lognormal relaxed molecular clock [27]. Skygrid coalescent tree priors were applied. Markov Chain Monte Carlo (MCMC) analyses were run for 100 million steps, with sampling every 1000 generations. Convergence and effective sample sizes (ESS) were assessed using Tracer v1.7.2. A maximum clade credibility (MCC) tree was generated using TreeAnnotator, discarding 10% burn‐in. Phylogenetic trees were visualized in R using the ggtree v4.0.1 package [28].

GenBank accession numbers of both reference sequences and those generated in this study are provided in Table S1.

### 2.4. Functional Analysis of the LCR (In Silico)

Complete LCR sequences from three representative HPV16 variants selected based on phylogeny and clinical diagnosis—LCR_18PRY (A1, no lesion), LCR_171PRY (D3, CIN3), and LCR_1058PRY (A4, cancer)—together with the HPV16 reference sequence (HPV16REF_PaVE; GenBank accession K02718), were analyzed for putative transcription factor binding sites (TFBS). Analyses were conducted using a multiple sequence alignment spanning the complete LCR region (nt 7157–7906 and 1–82).

TFBS prediction and comparative analyses were performed in R (R Core Team, 2025) using the packages *TFBSTools* and *JASPAR2022* [29, 30]. Position frequency matrices (PFMs) were retrieved from the JASPAR CORE vertebrates database and converted to position weight matrices (PWMs) for scanning [31]. A curated subset of biologically relevant transcription factors was selected for analysis, including CEBPB, ETS1, FOS, FOXA1, FOXA2, FOXA3, JUN, JUNB, JUND, MAFK, NFIL3, NFKB1, PHOX2A, RAX, RAX2, REL, RELA, RELB, SOX9, SOX10, SP1, SPIB, SRF, SRY, STAT1, STAT3, VAX1, and YY1.

TFBS scanning was performed using the *searchSeq()* function with a minimum relative profile score threshold of 85%, considering both DNA strands. Predicted binding sites were mapped along each sequence and compared across variants using custom R scripts. Additional data processing and visualization steps were performed using the packages *Biostrings*, *dplyr, stringr*, *purrr*, *ggplot2*, *patchwork*, *IRanges*, *ggrepel*, and *tibble* [20, 21, 23, 24, 26, 32–34].

Predicted TFBS were compared with those identified in the HPV16 reference sequence. Binding sites were classified as conserved, gained, or lost relative to HPV16REF_PaVE based on overlap in predicted binding coordinates. These in silico analyses were used to assess whether sequence variation within the LCR region could alter the TFBS landscape of the HPV16 variants analyzed.

### 2.5. Transcriptional Activity Assay

Three HPV16 variants (A1, A4, and D3 sublineages) were selected for functional analysis. The complete LCR (nt 7157–82) was amplified and cloned into a plasmid vector. Recombinant constructs were generated using *Hind*III and *Kpn*I restriction sites and cloned into pGL3‐Basic or precut pGL4.17 vectors (Promega). Constructs were confirmed by sequencing in two independent PCR products.

C33A cells (HPV‐negative human cervical carcinoma; ATCC HTB‐31) were cultured in DMEM supplemented with 10% fetal calf serum and antibiotics. Cells were seeded (4 × 10^6^ per dish) 24 h before transfection. Transfections were performed using 4 *μ*g of plasmid DNA and 1 *μ*g of pCMV‐*β*‐Gal (internal control for transfection efficiency), with FuGENE (Promega), according to the manufacturer′s protocol. After 48 h, cells were lysed, and luciferase and *β*‐galactosidase activities were quantified using Promega ONE‐Glo Luciferase and the *β*‐galactosidase enzyme assays systems, respectively. Relative luciferase activity was normalized to *β*‐galactosidase and total protein content. Each construct was tested in triplicate across nine independent experiments. All functional assays were conducted at the Instituto do Câncer do Estado de São Paulo (ICESP), Brasil, as part of training exchanges [35].

Luciferase assay results were visualized as bar plots with individual data points overlaid to illustrate experimental variability. Graphs were generated using GraphPad Prism Version 10.6.1 and are presented as mean ± standard deviation from nine independent experiments. Statistical significance was assessed using one‐way ANOVA followed by Bonferroni′s multiple‐comparison test.

### 2.6. Statistical Analysis

All statistical analyses were performed in R (R Core Team, 2025) [19]. Data processing and statistical testing were conducted using custom scripts and the packages *dplyr*, *purrr*, and *tibble* [21, 24, 26]. Mutation variables were coded as binary (presence/absence) for each nucleotide position. Associations between individual mutation sites and CIN2+ status were evaluated using Fisher′s exact test. Odds ratios (ORs) and 95% confidence intervals (95% CI) were calculated. To account for multiple comparisons, *p* values were adjusted using both Bonferroni and false discovery rate (FDR) methods. *p* values were adjusted across all tested mutation sites. Given the exploratory nature of the study and the limited sample size, mutation‐level associations were interpreted with caution.

Additionally, the association between HPV16 variant groups, A1–A3 (European) versus non‐A1–A3 variants (including A4, B, C, and D lineages), and CIN2+ status was evaluated using Fisher′s exact test.

### 2.7. Data Visualization and Graphical Representation

Graphical representations of mutation distribution and variant profiles were generated in R (R Core Team, 2025) using the package *ggplot2* [19, 32]. Custom scripts were developed to visualize nucleotide variation across the HPV16 LCR as a linear genomic map, integrating mutation frequency, variant classification, and associated clinical data.

Aligned sequences were reshaped into long format to enable position‐wise visualization of nucleotide variation across samples. Variants were grouped according to lesion status (<CIN2 and CIN2+) and HPV16 sublineage classification. Positions significantly associated with CIN2+ status (*p* < 0.05) were highlighted in the graphical output.

Data manipulation and transformation were performed using the R packages *dplyr*, *tidyr*, and *stringr* [21–23]. Custom color schemes were applied to represent HPV16 sublineages, and mutation patterns were visualized as presence/absence matrices with nucleotide annotation. Additional graphical customization, including formatted axis labels and text elements, was implemented using *ggtext* [36].

## 3. Results

Of the 58 HPV16‐positive cervical samples, complete high‐quality LCR sequences were successfully obtained from 44 samples. Only these samples were included in subsequent phylogenetic, mutation, and functional analyses, whereas samples that did not meet predefined quality criteria for full‐length sequence determination were excluded. Among the 14 excluded samples, 13 corresponded to women without CIN2+ lesions and one to a CIN2+ case. Importantly, clinical diagnosis was unknown during sequencing procedures and exclusion was based exclusively on sequence quality criteria (Table S2).

**Figure 1:**
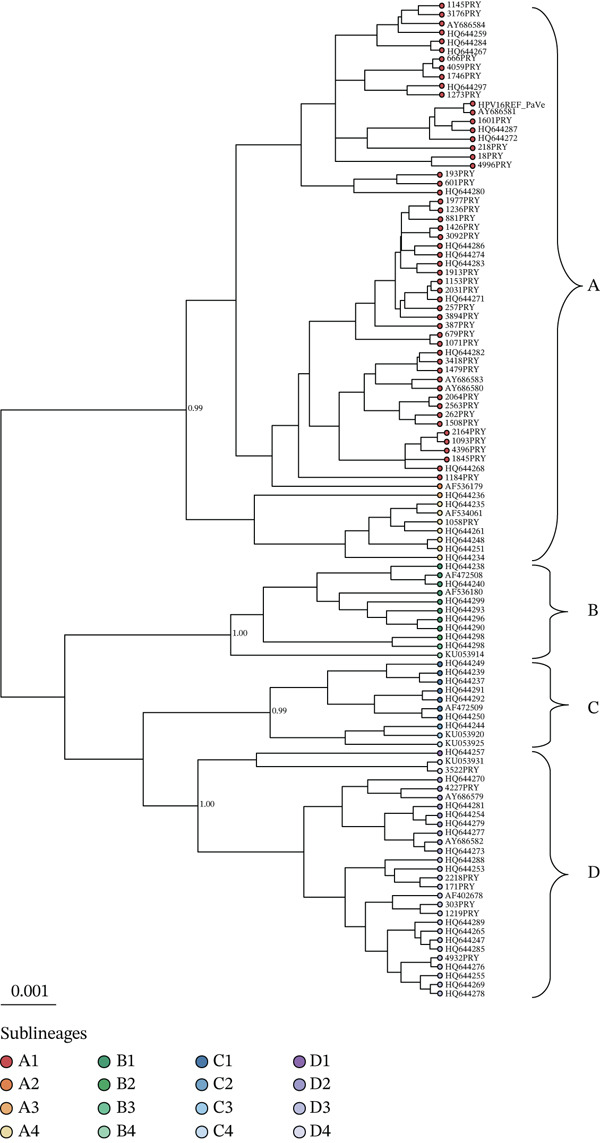
Phylogenetic analysis of the complete HPV‐16 LCR region based on 67 reference sequences and the 44 sequences of cervical samples belonging to Paraguayan women. Maximum clade credibility (MCC) tree of the HPV16 LCR region. Main lineages are indicated by right‐hand side braces. Sublineages are labeled in the names of the reference sequences. Samples belonging to the same sublineage share the same tip color. Paraguayan samples are shown in bold.

Most samples belonged to lineage A (37/44, 84.1%), particularly sublineage A1 (36/44, 81.8%), previously referred to as the European branch, followed by sublineage A4 (1/44, 2.3%), Asian branch, and others as part of lineage D (7/44, 15.9%), including sublineages D2 (1/44, 2.3%), D3 (5/44, 11.4%), corresponding to the Asian‐American branch, and D4 (1/44, 2.3%). All these non‐A1–A3 variants in this dataset were detected in women with CIN2+ (Table S2). Phylogenetic analysis based on the LCR region, incorporating 67 reference sequences and the 44 study samples (Table S1), is shown in Figure 1.

A total of 48 point mutations were identified across 47 nucleotide positions in the LCR region. Mutation frequencies ranged from 2% to 75%, with G7521A being the most frequent change, detected in 75% of the analyzed samples (Table S3).

The association between individual mutations and CIN2+ status was evaluated using Fisher′s exact test. Eleven mutations (A7233C, A7339T, C7394T, C7395T, A7485C, G7489A, C7669T, C7689A, C7764T, C7786T, and C7886G) showed nominal statistical significance (*p* < 0.05), several of which were exclusively or predominantly observed in CIN2+ cases, resulting in large or nonestimable ORs due to sparse data.

**Figure 2:**
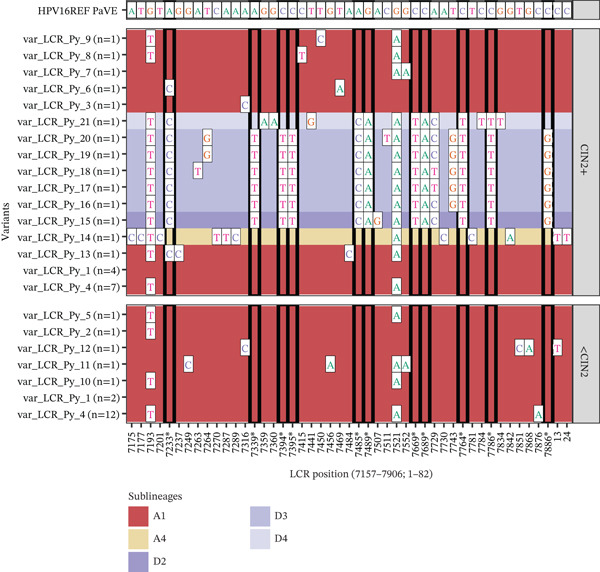
Distribution of nucleotide mutations across the HPV16 long control region (LCR) according to cervical lesion status. Linear representation of the complete HPV16 LCR (nucleotide positions 7157–7906 and 1–82), showing point mutations identified in cervical samples from Paraguayan women. Each vertical bar represents a nucleotide position with at least one mutation. Mutation frequencies are displayed separately for samples without cervical intraepithelial neoplasia grade 2 (<CIN2, *n* = 19) and for samples with cervical intraepithelial neoplasia grade 2 or worse (CIN2+, *n* = 25). Mutations showing nominal association with CIN2+ lesions (*p* < 0.05) are highlighted. These associations did not remain statistically significant after correction for multiple comparisons (Bonferroni or FDR). The genomic coordinates correspond to the HPV16 reference sequence (K02718; PaVE database).

These mutations were mainly detected in non‐A1–A3 variants (including A4 and D lineages). However, none of these associations remained statistically significant after correction for multiple comparisons (Bonferroni or FDR), indicating that these findings should be interpreted with caution (Figure 2).

**Figure 3:**
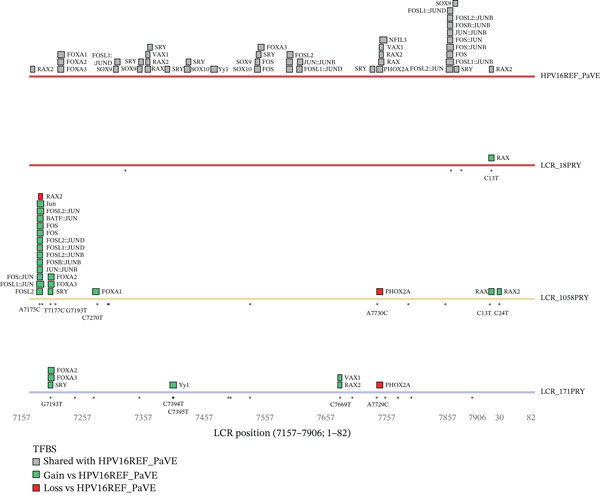
Predicted transcription factor binding sites in the HPV16 reference LCR and representative Paraguayan variants. Predicted TFBS were identified using TFBSTools with JASPAR2022 CORE vertebrate matrices and a minimum relative profile score threshold of 85%. Shared sites with HPV16REF_PaVE are shown together with predicted gain and loss events in LCR_18PRY (A1, no lesion), LCR_171PRY (D3, CIN3), and LCR_1058PRY (A4, cancer). Asterisks indicate nucleotide differences relative to the reference sequence. TFBS predictions are computational and should be interpreted as hypothesis‐generating.

In silico TFBS analysis identified 196 predicted binding sites across all sequences, of which 29 corresponded to gain and loss events relative to the HPV16 reference sequence (HPV16REF_PaVE) (Figure 3).

The LCR_18PRY sequence (A1, no CIN2+ lesion) showed a TFBS profile largely conserved with the reference, with only one predicted gain event. In contrast, LCR_171PRY (D3, CIN3) displayed six gains, including motifs associated with FOXA2, FOXA3, SRY, YY1, RAX2, and VAX1, and loss of PHOX2A.

The LCR_1058PRY variant (A4, cancer) exhibited the most divergent profile, with 19 gains and two losses. Notably, multiple gained motifs clustered within the 7170–7181 region and corresponded to AP‐1‐related transcription factors (JUN/FOS), along with additional gains involving FOXA1, FOXA2, FOXA3, SRY, RAX, and RAX2. Two loss events (RAX2 and PHOX2A) were also identified.

Table S4 shows the sequences of the HPV16 variants whose transcriptional activity was measured.

**Figure 4:**
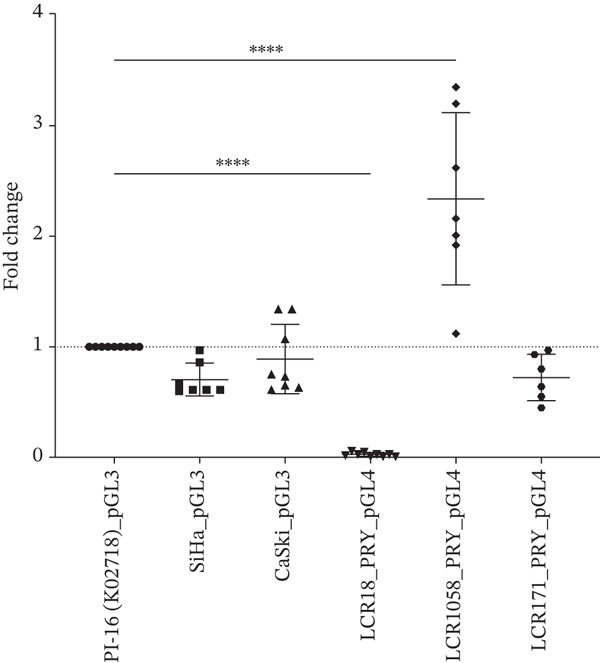
Transcriptional activity of HPV16 LCR variants measured by luciferase assay. C33A cells were transiently transfected with luciferase reporter constructs containing the complete HPV16 long control region (LCR) from different variants. Transcriptional activity was normalized to *β*‐galactosidase activity and total protein content and is expressed as fold change relative to the reference construct PI‐16 (K02718), which was arbitrarily set to 1 (dotted line). Each symbol represents an independent experiment, and horizontal bars indicate mean ± standard deviation from nine independent experiments. Statistical analysis was performed using one‐way ANOVA followed by Bonferroni′s multiple‐comparison test. ∗∗∗∗*p* < 0.0001.

In vitro assessment of transcriptional activity revealed significant differences among HPV16 LCR constructs (Figure 4).

Compared with the reference construct PI‐16 (K02718, A1), the Bonferroni‐adjusted multiple‐comparison test indicated that LCR_1058PRY (A4, cancer) exhibited significantly higher transcriptional activity (mean fold change 2.34 ± 0.78; *p* < 0.0001), whereas LCR_18PRY (A1, NSIL) showed markedly reduced promoter activity (0.03 ± 0.02; *p* < 0.0001).

In contrast, no statistically significant differences were observed between the reference construct and SiHa (A1), CaSki (A1), or LCR_171PRY (D3, CIN3) after correction for multiple comparisons.

Given that no individual mutation remained statistically associated with CIN2+ lesions after correction for multiple comparisons, we further investigated whether broader HPV16 variant groupings correlated with lesion severity. The subsequent analysis revealed an exploratory association between non‐European variants (A4, D2, D3, and D4) and CIN2+ lesions. These variants were exclusively detected in women with CIN2+, whereas European variants (A1–A3) were present in both <CIN2 and CIN2+ groups (Fisher′s exact test, OR = ∞, 95*%*CI = 1.58–∞, *p* = 0.0065; Table 1).

**Table 1:** Frequency of HPV16 variants identified by sequencing the complete LCR region from 44 cervical samples from Paraguayan women according to gynecological diagnosis.

Lineage	Sublineage	Branch	LCR_Variant	<CIN2	CIN2+
				*n* (%)	*n* (%)
Total				19	25
European variants					
A	A1	E	var_LCR_Py_1	2 (10.5)	4 (16)
A	A1	E	var_LCR_Py_2	1 (5.3)	0 (0)
A	A1	E	var_LCR_Py_3	1 (5.3)	0 (0)
A	A1	E	var_LCR_Py_4	12 (63.2)	7 (28)
A	A1	E	var_LCR_Py_5	0 (0)	1 (4)
A	A1	E	var_LCR_Py_6	0 (0)	1 (4)
A	A1	E	var_LCR_Py_7	0 (0)	1 (4)
A	A1	E	var_LCR_Py_8	0 (0)	1 (4)
A	A1	E	var_LCR_Py_9	1 (5.3)	0 (0)
A	A1	E	var_LCR_Py_10	0 (0)	1 (4)
A	A1	E	var_LCR_Py_11	1 (5.3)	0 (0)
A	A1	E	var_LCR_Py_12	0 (0)	1 (4)
A	A1	E	var_LCR_Py_13	1 (5.3)	0 (0)
			Subtotal	19 (100)	17 (68)
Non‐European variants^a^					
A	A4	As	var_LCR_Py_14	0 (0)	1 (4)
D	D2	AA	var_LCR_Py_15	0 (0)	1 (4)
D	D3	AA	var_LCR_Py_16	0 (0)	1 (4)
D	D3	AA	var_LCR_Py_17	0 (0)	1 (4)
D	D3	AA	var_LCR_Py_18	0 (0)	1 (4)
D	D3	AA	var_LCR_Py_19	0 (0)	1 (4)
D	D3	AA	var_LCR_Py_20	0 (0)	1 (4)
D	D4		var_LCR_Py_21	0 (0)	1 (4)
			Subtotal	0 (0)	8 (32)

Abbreviations: AA, Asian–American; As, Asian; CIN2, cervical intraepithelial neoplasia grade 2; E, European.

^a^These variants were exclusively detected in women with CIN2+, compared with European variants (A1–A3) that were present in both <CIN2 and CIN2+ groups (Fisher′s exact test, OR = ∞, 95*%*CI = 1.58–∞, *p* = 0.0065). The infinite odds ratio observed resulted from the absence of non‐A1–A3 variants in the <CIN2 group.

## 4. Discussion

The distribution of HPV16 variants identified in this study is consistent with previous research conducted in Paraguay by Mendoza et al., which found that the majority of sequences belonged to the A1–A3 (European) sublineage, followed by the D (Asian–American) lineage variants. However, a notable contribution of the current work is the identification, for the first time in Paraguayan women, of a variant from the A4 (Asian) sublineage, detected in a woman with cancer, which showed the greatest transcriptional activity compared with the reference HPV 16 variant. Unlike the study by Mendoza et al., no variants from B and C (African) lineages were detected in this sample set [37].

Comparative data from neighboring countries also reflect similar lineage patterns. In Argentina, Badano et al. reported the predominance of lineage A followed by D, whereas Oliveira et al. found variants from lineages A, B, and D among Brazilian women. These findings support the notion of regional diversity in HPV16 variant distribution, with a predominance of A1–A3 and D sublineages in South America [38, 39].

We observed that no individual mutation remained statistically associated with CIN2+ lesions after correction for multiple comparisons. These findings are not consistent with previous reports by Kammer et al. and Mirabello et al., which described nucleotide substitutions in D lineage variants associated with increased transcriptional activity compared with A1/A2 (European) variants [40, 41]. This difference may be due to the limited sample size of the present study.

Additionally, our functional analysis revealed that the LCR sequence of the A4 sublineage variant exhibited the highest transcriptional activity. This is consistent with the study by Pientong et al., which demonstrated that A4 (Asian) sublineage variants possess higher promoter activity than both A1–A3 (European) and D (Asian–American) lineage counterparts [42]. However, it is important to note that this finding is based on a single A4 sample and should therefore be interpreted with caution.

In parallel, in silico analysis using the JASPAR database indicated that the A4 variant displayed the most divergent predicted transcription factor binding profile relative to the reference sequence, with a higher number of predicted gain/loss events, particularly involving AP‐1‐related transcription factors (JUN/FOS) and members of the FOXA family. Previous studies have suggested that sequence variability within the HPV16 LCR can modify the transcription factor binding landscape and may be associated with differences in promoter activity [14, 43].

Notably, several predicted gain events in the A4 sequence were located within regulatory regions previously implicated in early promoter activation. AP‐1 family members, including JUN and FOS, have been associated with HPV transcriptional regulation and may represent potentially biologically relevant motifs in this context [44]. However, these observations are based on computational predictions and should be interpreted with caution. Although the A4 variant also exhibited the highest transcriptional activity in the luciferase assay, the present data do not allow a direct causal relationship to be established between specific TFBS changes and promoter activity. Further functional studies, including targeted mutagenesis of predicted TFBS and knockdown of relevant transcription factors in luciferase assay models, will be necessary to establish direct mechanistic links between specific LCR sequence changes and transcriptional activity.

Variant‐specific patterns, such as the exclusive prediction of VAX1‐associated motifs in LCR_171PRY and JUN‐related motifs in LCR_1058PRY, further suggest differences in the predicted regulatory landscape among variants; however, their biological significance remains to be experimentally validated.

Importantly, we observed an exploratory association between non‐A1–A3 (non‐European) variants and CIN2+ status, with these variants being exclusively detected in CIN2+ samples within this dataset. The grouping of variants into A1–A3 (European) and non‐A1–A3 (non‐European) categories was based on a commonly used phylogeographic classification in previous studies, where A1–A3 variants are considered of European origin and A4, B, C, and D sublineages are grouped as non‐European. This approach has been widely applied to investigate differences in oncogenic potential between variant groups and allows comparability with existing literature. However, this grouping combines biologically distinct sublineages, and the limited number of non‐A1–A3 variants in our dataset precluded meaningful stratified analyses by individual sublineages. This finding is consistent with previous epidemiological evidence suggesting that non‐A1–A3 (non‐European) HPV16 variants, particularly those from A4 and D lineages, may be associated with increased oncogenic potential. Studies by Schiffman et al. and Mirabello et al. demonstrated that sublineages A4, D2, and D3 are associated with a higher risk of cervical lesions compared with A1/A2, with A4 showing the strongest association with cancer risk [41, 45, 46]. In contrast, B lineage variants have been associated with a lower risk of CIN3 [41]. These findings, together with our results, support the possibility that non‐A1–A3 HPV16 variants may differ in their biological behavior, although further studies are needed to confirm these observations, particularly in highly admixed populations where variant diversity may be greater. In fact, as highlighted by Mirabello et al., host‐related factors, including race and ethnicity, may also influence the natural history of HPV16 infections and should be considered in future research.

Recent studies worldwide have further expanded the understanding of HPV16 variability beyond the LCR. Bletsa et al. highlighted that polymorphisms in early genes, particularly *E6* and *E7*, may have functional consequences that complement the effects of LCR mutations [47]. In fact, Sichero et al. showed that human keratinocytes immortalized by different HPV‐16 *E6* and *E7* variants diverge in their biological properties associated with cancer hallmarks [48].

Although our study focused exclusively on the LCR, previous studies have shown that structural genes such as *L1* and *L2* exhibit genetic diversity across different geographic regions [49]. Although the functional consequences of these variations remain uncertain, their location within conserved or structurally relevant domains may suggest potential immunological implications. A recent analysis by van Eer et al. compared HPV16 sequences from vaccinated and unvaccinated young women, identifying minor genetic differences and discussing the potential impact of immunization on viral diversity. These findings highlight the need for continued genomic surveillance in diverse populations, particularly where non‐A1–A3 (non‐European) variants are prevalent, to ensure long‐term effectiveness of vaccination [1].

It is important to note that complete LCR sequences were obtained for 44 of the 58 HPV16‐positive samples initially included. The exclusion of samples that did not meet quality criteria for full‐length sequencing may have introduced selection bias and could limit the generalizability of the findings, particularly regarding variant distribution and lesion status. Notably, most excluded samples corresponded to women without CIN2+ lesions, which may have influenced the observed distribution of HPV16 variants across lesion groups. Nevertheless, the fact that this study focused on the LCR region may constitute a potential limitation, as this restricts our ability to capture the full spectrum of genomic variability, including mutations in early and late genes that may influence viral persistence and oncogenic potential. Additionally, the limited number of non‐European variants included in this study may affect the robustness and generalizability of the observed associations. However, several studies have demonstrated that the LCR, particularly when analyzed in combination with genes such as *E6*, can provide sufficient phylogenetic resolution for accurate classification of HPV16 variants. Cornet et al. showed a strong concordance between phylogenies based on *E6* and LCR and those based on whole‐genome sequences, supporting the use of these regions for lineage and sublineage assignment. Similarly, Bletsa et al. and Mirabello et al. emphasized the functional relevance of LCR and early gene variability, noting that differences in transcriptional activity and oncogenic risk between sublineages can often be traced to mutations within these regions. Furthermore, Ou et al. demonstrated that a limited number of genomic positions, many of which are located in LCR and early genes, are sufficient to classify variants with high accuracy, reinforcing the value of targeted sequencing approaches for molecular epidemiology purposes. Future studies combining targeted and whole‐genome approaches, while incorporating host and immunological factors, will be crucial to deepening our understanding of the factors that shape HPV16 pathogenesis and its clinical implications [41, 47, 50, 51].

In addition, the functional analyses performed in this study do not allow direct validation of the mechanistic contribution of individual TFBS alterations to promoter activity. Future studies incorporating targeted mutagenesis and transcription factor modulation approaches will be important to confirm the biological relevance of the predicted TFBS changes identified in the A4 and D lineage variants.

## 5. Conclusion

This study provides novel insights into the genetic variability and functional behavior of HPV16 variants circulating among Paraguayan women. The predominance of lineage A1 is consistent with previous reports from the region; however, the detection of an A4 sublineage variant—reported here for the first time in Paraguay—highlights the growing genetic diversity of HPV16 that could be associated with more recent population migration in this country. Both in silico and experimental analyses showed that the A4 variant exhibited the most divergent predicted transcription factor binding profile and the highest transcriptional activity among the variants evaluated.

These findings reinforce the potential importance of intratype HPV16 diversity and suggest that specific non‐A1‐A3 variants, especially A4, may differ in their biological behavior. However, the observations related to the A4 variant are based on a single isolate and should therefore be interpreted with caution until validated in larger studies. Further studies incorporating larger longitudinal cohorts, whole‐genome sequencing approaches, host genetic factors, and vaccinated populations will be necessary to clarify the biological and clinical relevance of HPV16 variant‐specific differences.

## Author Contributions

Pamela Mongelós and Magaly Martínez contributed equally to this work.

## Funding

This work was funded by grants from the National Council for Science and Technology (CONACYT PINV 18‐896), Asunción‐Paraguay, and from the International Agency for Research on Cancer, World Health Organization, Lyon‐France (IARC‐WHO CRA PRI/18/05).

## Disclosure

M.R. is identified as personnel of the International Agency for Research on Cancer/World Health Organization. The authors alone are responsible for the views expressed in this article, which do not necessarily represent the decisions, policies, or views of the International Agency for Research on Cancer/World Health Organization. M.A. is a former staff member of the World Health Organization. The authors alone are responsible for the views expressed in this publication, which do not necessarily represent the views, decisions, or policies of the institutions. A.B. was supported in part by an appointment to the US National Cancer Institute (NCI) Research Participation Program administered by the Oak Ridge Institute for Science and Education (ORISE) through an interagency agreement between the U.S. Department of Energy (DOE) and the National Institutes of Health. ORISE is managed by ORAU under DOE contract number DESC0014664. All opinions expressed in this paper are those of the authors and do not necessarily reflect the policies and views of NIH, NCI, DOE, or ORAU/ORISE. A.B. was supported in part by the Intramural Research Program of the National Institutes of Health (NIH). The contributions of the NIH author(s) were made as part of their official duties as NIH federal employees, were in compliance with agency policy requirements, and were considered Works of the United States Government. However, the findings and conclusions presented in this paper are those of the author(s) and do not necessarily reflect the views of the NIH or the US Department of Health and Human Services.

## Ethics Statement

This study was conducted in accordance with the principles outlined in the Declaration of Helsinki. In ESTAMPA, all participants provided written informed consent for the future use of their samples in research. All collected specimens, including exfoliated cervical cells, were anonymized to ensure participant confidentiality. For this study, the ESTAMPA PI in Paraguay selected and linked the samples to epidemiological data and disease status using a coded dataset. Therefore, for this study, the researchers had access to the data collected under coded identifiers after obtaining the approval of the Scientific and Ethics Committees of the project mentioned above (Code P29/2018) on date 22/06/2020.

## Conflicts of Interest

The authors declare no conflicts of interest.

## Supporting Information

Additional supporting information can be found online in the Supporting Information section.

## Supporting information


**Supporting Information 1** Table S1: Alignment of the 67 HPV 16 reference sequences and the 44 sequences of the LCR region of Paraguayan women positive for HPV16 from the ESTAMPA study.

**Supporting Information 2** Table S2: HPV 16 LCR sequences from cervical samples of Paraguayan women participating in the ESTAMPA study.

**Supporting Information 3** Table S3: Frequency and statistical association of HPV16 LCR mutations with CIN2+ status in Paraguayan women.

**Supporting Information 4** Table S4: Sequence variability of the long control region and transcriptional activity of different human papillomavirus type 16 (HPV‐16) molecular variants.

## Data Availability

The data supporting the findings of this study are available within the article and its Supporting Information. Additional data are available from the corresponding author upon reasonable request.
